# Impact of evening blue light exposure timing on sleep, motor, and cognitive performance in young athletes with intermediate chronotype

**DOI:** 10.5114/biolsport.2025.146787

**Published:** 2025-01-20

**Authors:** Mohamed Abdelkader Souissi, Chadha Gouasmia, Ismail Dergaa, Jihed Faleh, Omar Trabelsi, Katja Weiss, Thomas Rosemann, Wissem Dhahbi, Nizar Souissi, Beat Knechtle

**Affiliations:** 1 Physical Activity, Sport and Health Research Unit, UR18JS01, National Observatory of Sport, Tunis, Tunisia; 2 High Institute of Sport and Physical Education Gafsa, Gafsa University, Gafsa, Tunisia; 3 Primary Health Care Corporation (PHCC), Doha, Qatar; 4 High Institute of Sport and Physical Education of El Kef, University of Jendouba, El Kef, Tunisia; 5 Institute of Primary Care, University of Zurich, Zurich, Switzerland; 6 Qatar Police Academy, Police College, Training Department, Doha, Qatar; 7 High Institute of Sport and Physical Education of Ksar Said, University of Manouba, Manouba, Tunisia; 8 Medbase St. Gallen Am Vadianplatz, St Gallen, Switzerland

**Keywords:** Circadian rhythm, Digital screen exposure, Melatonin inhibition, Neurobehavioral efficacy, Psychomotor vigilance, Sleep hygiene, Sport-specific competencies, Adolescents

## Abstract

Evening exposure to blue light has been shown to negatively affect sleep patterns and cognitive function. However, the effect of the specific timing of blue light exposure on motor performance and sleep quality in adolescent athletes remains unclear. This study aimed to investigate the impact of evening blue light exposure (BLE) timing on sleep quality, motor performance, and cognitive function in young athletes with intermediate chronotypes. Sixteen male adolescent athletes (age: 15.11 ± 0.92 years, body height: 168.25 ± 7.09 cm, body-mass: 59.49 ± 5.63 kg) participated in a randomized, crossover study with four conditions: BLE from (i) 7: 30–9: 00 PM, (ii) 9: 00–10: 30 PM, (iii) 10: 30 PM-12: 00 AM, and (iv) control (no BLE). Sleep quality and quantity were evaluated using the Spiegel and Vis-Morgen questionnaires. Selective attention was assessed using the Bells Test, while motor function was tested by dart-throwing accuracy and movement duration. ANOVA indicated substantial main effects of BLE timing on sleep metrics, motor performance, and cognitive function. Total sleep duration was markedly shorter in the 9: 00–10: 30 PM and 10: 30 PM-12: 00 AM conditions relative to the control (p < 0.001, d = 0.54 [medium] and d = 0.87 [large], respectively) and the 7: 30–9: 00 PM condition (p < 0.05, d = 0.28 [small] and p < 0.001, d = 0.56 [medium], respectively). Dart-throwing accuracy was significantly lower in the 9: 00–10: 30 PM and 10: 30 PM-12: 00 AM conditions versus the control (p < 0.002, d = 0.77 [medium]) and p < 0.001, d = 1.41 [large], respectively). Movement duration was significantly longer in these conditions compared to the control (p < 0.001, d = 1.75 [large] and d = 1.51 [large], respectively) and 7: 30–9: 00 PM condition (p < 0.01, d = 1.38 [large] and p = 0.002, d = 1.17 [large], respectively). Selective attention was significantly lower in the 9: 00–10: 30 PM and 10: 30 PM-12: 00 AM conditions compared to the control (p = 0.003, d = 0.66 [medium] and p < 0.001, d = 0.91 [large], respectively). Evening BLE, especially after 9: 00 PM, adversely affects sleep quality, motor performance, and cognitive function in young athletes. These findings underscore the necessity of reducing BLE in the evening to enhance sports performance, optimize training and recovery, and facilitate motor learning for skill development.

## INTRODUCTION

The widespread availability of internet connectivity in various parts of the world has accelerated the rapid spread of technical instruments, significantly influencing modern ways of living [1]. Remarkably, electronic screens have seen an unprecedented increase among children [2], adolescents [3], and adults [4] in recent years. Indeed, Meng et al. [5] found that, in the past two years, the global prevalence of smartphone addiction was 34.5% among university and high school students. The advent of digital immersion has sparked worries over its possible effects on human physiology and performance, specifically regarding sleep patterns and cognitive function [6]. These electronic screens facilitate individuals’ lives and are frequently used in various contexts for communication [7], entertainment [8], and education [9].

Although recent studies have shown that video technology is effective in improving teaching [10] and training [11] outcomes compared to traditional techniques, it is important to investigate the potential negative effects of prolonged screen exposure. These advantages are attributed to improved information processing [12] and the development of students’ knowledge, skills, and attitudes [10]. Furthermore, according to previous studies, exposure to electronic screens seems to stimulate brain networks associated with various aspects of cognitive performance. Working memory, a crucial cognitive function, could particularly benefit from this exposure. Working memory involves various cognitive processes that actively maintain information in memory to guide decision-making [13].

Nevertheless, the effects of screen exposure can be considerably influenced by the timing, especially if it happens during the evening hours [14]. The majority of studies were carried out during daylight hours, indicating that exposure to screens increases wakefulness [14], psychomotor vigilance [18], and evening mood. Chang et al. [19] observed that reading electronic books in the evening diminishes alertness the following morning compared to reading printed books. Indeed, researchers have linked this drop in alertness to interrupting the body’s natural circadian cycle caused by reading electronic books at night.

Research has demonstrated that the blue light emitted by electronic screens can inhibit the generation of melatonin, prolong the time it takes to fall asleep and disturb the body’s natural sleep-wake cycle, which may negatively impact both the quality and amount of sleep [20–23]. Sleep disruptions have a detrimental effect on physical performance, the process of recovery, and participation in competitive sports [20, 21]. Numerous studies have demonstrated that optimal sleep is critical for athletic recovery, motor learning, and cognitive functioning, which are all essential for peak performance in competitive sports [21, 24]. This is particularly concerning for athletes whose performance and recovery depend heavily on optimal sleep patterns [25]. Inadequate sleep has a negative impact on the storage of muscle glycogen, stress levels, and sprint performance. Excessive exposure to blue light before bedtime has been shown to have an adverse effect on cognitive abilities [25] and academic accomplishment [22, 23], which may impact memory and focus [26, 27]. Engaging in television viewing and video game playing has a detrimental effect on academic achievement, particularly among adolescents [28]. Similarly, using media before going to bed might result in difficulties with sleep [29].

To the best of our knowledge, no studies have thoroughly examined the impact of blue light exposure at various night-time hours on the quality of sleep, motor performance, and cognitive function in adolescent athletes with intermediate chronotypes. Intermediate chronotypes were selected due to their prevalence among adolescents and because they exhibit flexible sleep-wake patterns, making them an ideal population for investigating the impact of external factors such as blue light exposure on performance. Understanding how blue light affects this specific group can provide valuable insights into optimizing sleep and performance strategies tailored for adolescent athletes. Hence, this study aimed to clarify the chronological connection between exposure to blue light in the evening and the subsequent quality of sleep, motor performance, and cognitive function in young athletes.

## MATERIALS AND METHODS

### Participants

The G*Power software was used beforehand to determine the minimum required sample size, following the procedures recommended by Kang et al. [30]. The significance level (α) was set at 0.05 and the statistical power at 0.95. Based on a study with a similar design and after thorough discussions among the authors, we estimated that the effect size would be approximately 0.45. Thus, to achieve the desired statistical power, we determined that a minimum of 12 participants would be sufficient, effectively reducing the risk of encountering a type II statistical error.

Twenty-eight young athletes were identified as potential participants for this research. Ten were excluded before the start of the study due to not meeting the inclusion criteria (detailed below), and two did not complete the full experimental protocol.

Sixteen healthy male adolescent athletes (age: 15.11 ± 0.92 years; body height: 168.25 ± 7.09 cm; body mass: 59.49 ± 5.63 kg; body mass index: 21.0 ± 1.5 kg · m^−2^) voluntarily participated in this study. The study recruited participants from nearby sports clubs who had been consistently involved in athletic training for a minimum of two years, before the study. Eligibility criteria included: (i) an intermediate chronotype, with a score on the Morningness-Eveningness Questionnaire (MEQ) between 42 and 58 [31]; (ii) no history of musculoskeletal or neurological disorders, nor any injuries or surgery in the six months preceding the tests that could affect their physical abilities; (iii) no visual problems; (iv) no experience in shooting sports; and (v) no sleep problems (Pittsburgh Sleep Quality Index score < 5).

Participants and their parents or legal guardians were informed about the testing procedures and experimental conditions before providing their written consent. Written consent from the participants, as well as their parents or legal guardians, was obtained. The study protocol adhered to the principles outlined in the Declaration of Helsinki for research involving humans and received approval from the ethics committee of the Faculty of Medicine of Sfax, with reference number 49/24. It also complied with the ethical and procedural requirements for the conduct of sports medicine and exercise science research [32].

### Experimental procedure

To minimize learning effects throughout the experiment, participants were thoroughly familiarized with the study procedures and tests during the previous week. The experimental protocol included four distinct conditions, each involving a specific time of evening exposure to blue light: (i) exposure from 7: 30 PM to 9: 00 PM, (ii) exposure from 9: 00 PM to 10: 30 PM, (iii) exposure from 10: 30 PM to 12: 00 AM, and (iv) a control condition with no exposure. The sunset during this study period was approximately 7: 30 PM. Bedtime was set at 12: 00 AM and wake-up time at 8: 30 AM. All sessions were performed indoors and at the same time of the day to minimize the effects of diurnal variations in the measured variables [33]. Sessions were conducted three days away from the full moon days to avoid the effect of circa-lunar variation on the measured parameters [34]. During each blue light exposure condition, participants were required to watch the same video content displayed on an 11-inch screen tablet (Lenovo Yoga Tab 11). The video content was standardized across all conditions to ensure consistency and minimize variability. It was selected for its neutral entertainment value, avoiding any elements that could engage participants cognitively or emotionally. The tablet’s blue light was quantified using a spectroradiometer (Model PR-655, Photo Research, Chatsworth, CA, USA). The measured blue light had a peak wavelength of 460 nm and an intensity of 30 μW · cm^−2^ when viewed from a distance of 50 cm. This content, recorded before each experimental night, was of an entertainment nature.

In this study, we used a randomized crossover design where each participant was exposed to all experimental conditions but in a random order. Each night, four participants were assigned to conditions different from the others, and then the conditions were alternated. The participants’ parents were responsible for supervising their sons during this study. They were required to provide the tablets at the specified time and remove them at the time requested by the experimenters according to the study conditions. Parents also had to report any situation where their sons did not adhere to the exposure conditions. Tests were scheduled in the morning between 10: 00 AM and 12: 00 PM. The testing sessions were conducted in a randomized and balanced order. Each session successively included a dart throwing test to evaluate precision and movement duration and a selective attention test.

Additionally, the subjective quality and quantity of sleep were assessed after each night using the Vis-Morgen and Spiegel Sleep Questionnaire. The assessment of chronotype was conducted using the MEQ. The MEQ was chosen to ensure that all participants had intermediate chronotypes, as these individuals are less likely to experience extreme circadian phase shifts, allowing for a more controlled examination of the timing of blue light exposure on performance. The experimental conditions were separated by a 72-hour washout period to minimize potential carryover effects and neutralize learning effects. Before each experimental night or testing session, several preventive measures were put in place. Participants were encouraged to avoid any intense physical activity in the 24 hours preceding each testing session and to maintain a regular sleep schedule. Moreover, they were prohibited from consuming any known stimulant, such as caffeine, or any depressant, such as alcohol, which could potentially influence their alertness either positively or negatively [11].

### Data collection

#### Vis-Morgen Sleep Questionnaire

The Vis-Morgen Sleep Questionnaire includes a visual analogue scale that measures the participant’s energy upon waking (feeling of freshness). This scale scores from 0 (low) to 10 (very high) [35]. The questionnaire also includes (i) a question about sleep onset latency and (ii) a question about total sleep time. The Vis-Morgen and Spiegel sleep questionnaires have been validated for use in adolescent populations and demonstrate good test-retest reliability (*r* = 0.85 and *r* = 0.78, respectively) [36].

### Spiegel Sleep Questionnaire

The subjective quality of sleep was measured using the Spiegel Sleep Questionnaire [37]. This test includes six questions designed to assess sleep quality. It generates a score between 0 and 30, with a higher score indicating better sleep quality [37].

### Dart-throwing accuracy

The task performed in this study involved dart throwing. Each participant had to attempt to throw six metal darts, each weighing 25 g and measuring 15 cm in length, one by one, at a dartboard. This dartboard featured nine concentric circles, each measuring 2.54 cm in width. According to the World Darts Federation (WDF) rules, the dartboard was set up with the centre at a height of 1.73 m (5 feet 8 inches) above the floor. The throwing line, also known as the oche, was marked on the floor at a distance of 7 feet 9¼ inches (2.37 m) from the face of the dartboard.

For scoring, each circle was assigned a numerical value, starting at 10 points for the central circle and gradually decreasing by 1 point as one moved outward, with the outermost circle having a value of 1 point and 0 points outside this circle. Thus, the score for each throw could range from zero to ten. The average score for a set of six dart throws was calculated by dividing the total score by six. Dart-throwing skills were evaluated in each session.

### Movement duration

Each dart-throwing trial was filmed and recorded by a Sony HXR-MC2500 camera (50 frames per second). The movement duration was calculated using Kinovea software (version 0.8.15). The initial time was marked as the moment when the participant took the dart with their dominant hand from their non-dominant hand, and the final time was marked as the moment the dart left the fingers towards the target. The average movement duration was calculated by dividing the sum of these six durations by six.

### Selective attention

The Bells Test, a validated tool for assessing sustained and selective attention (test-retest reliability: *r* = 0.76), was administered as described by Gauthier et al. [38]. This test consists of four distinct sheets, each containing 35 bells among a total of 280 distractor elements (such as houses, trees, fish, and horses). All these images are of the same size and orientation, printed in black on an 11 × 8.5-inch page. The participants have the page placed in front of them, aligned with their midline. The task assigned to them is to locate and mark only the bells within a limited time of 2 minutes per sheet. We then calculated the number of bells identified during the first 30 seconds on each sheet, constituting the selective attention score.

### Statistical analyses

**FIG. 1:**
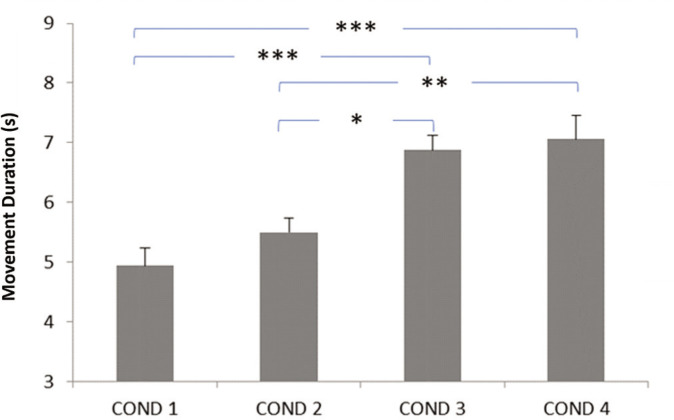
Impact of evening blue light exposure timing on movement duration. Later exposure periods (particularly after 9: 00 PM) significantly increased movement duration compared to no exposure or early evening exposure, suggesting impaired motor performance. COND 1: no blue light exposure; COND 2: blue light exposure from 7: 30 PM to 9: 00 PM; COND 3: blue light exposure from 9: 00 PM to 10: 30 PM; COND 4: blue light exposure from 10: 30 PM to 12: 00 AM; (s): seconds. * Significant difference at *p* < 0.01; ** Significant difference at *p* = 0.002; *** Significant difference at *p* < 0.001.

**FIG. 2:**
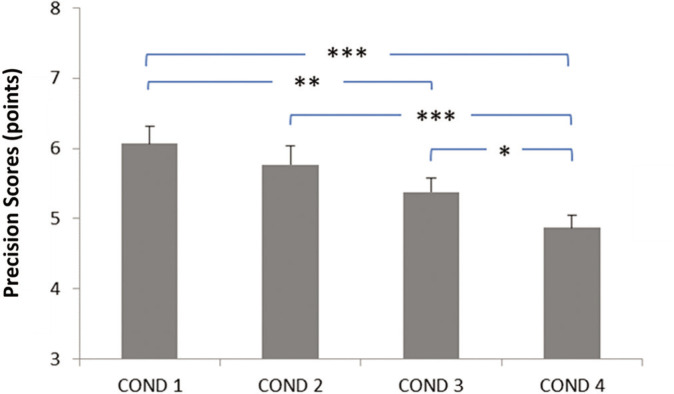
Effect of evening blue light exposure timing on dart-throwing precision. Progressive deterioration in precision scores was observed with later exposure times, with most pronounced deficits occurring during late-night exposure (10: 30 PM-12: 00 AM). COND 1: no blue light exposure; COND 2: blue light exposure from 7: 30 PM to 9: 00 PM; COND 3: blue light exposure from 9: 00 PM to 10: 30 PM; COND 4: blue light exposure from 10: 30 PM to 12: 00 AM. * Significant difference at *p* < 0.05; ** Significant difference at *p* = 0.002; *** Significant difference at *p* < 0.001.

**FIG. 3:**
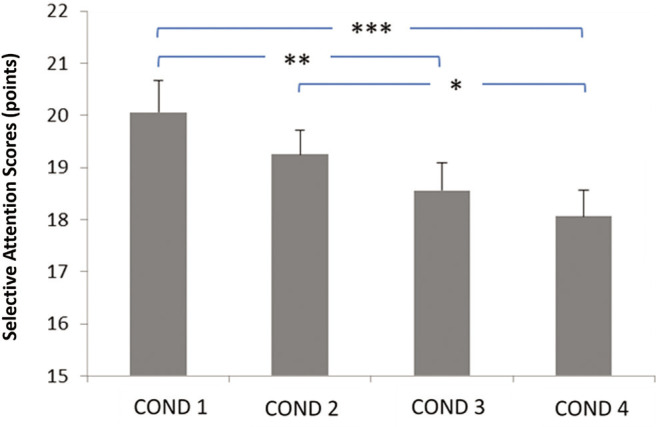
Relationship between evening blue light exposure timing and selective attention performance. Later exposure times were associated with progressive decline in attention scores, particularly critical for children’s daily cognitive functioning. COND 1: no blue light exposure; COND 2: blue light exposure from 7: 30 PM to 9: 00 PM; COND 3: blue light exposure from 9: 00 PM to 10: 30 PM; COND 4: blue light exposure from 10: 30 PM to 12: 00 AM. * Significant difference at *p* < 0.05; ** Significant difference at *p* = 0.003; *** Significant difference at *p* < 0.001.

Data are presented as means accompanied by (i) their standard deviations (± SD) in Table 1 and (ii) their standard errors (± SE) in Figures 1, 2, and 3. Statistical analysis was performed using Statistica 10 software (StatSoft, Krakow, Poland). The normality of the data was verified using the Shapiro-Wilk test, and the data showed a normal distribution. The main effect of the condition of evening blue light exposure timing (No exposure, Exposure from 7: 30 PM to 9: 00 PM, Exposure from 9: 00 PM to 10: 30 PM, and Exposure from 10: 30 PM to 12: 00 AM) was evaluated via a one-way repeated measures ANOVA on sleep, cognitive, and motor parameters. In case of significant main effects, a Bonferroni post-hoc test was applied for pairwise comparisons of the conditions. Effect sizes were determined using partial eta squared (ηp^2^) for the repeated measures ANOVA and interpreted according to Cohen’s classification (trivial effect sizes < 0.2, small < 0.5, moderate < 0.8, and large ≥ 0.8). The threshold for statistical significance was set at *p* < 0.05.

**TABLE 1:** Variation of sleep parameters by timing of evening blue light exposure

Condition	COND 1	COND 2	COND 3	COND 4	P
Total Sleep Time (minute)	465.5 ± 42.19	455.56 ± 52.12	442 ± 43.49*©	428.5 ± 42.57*©¥	< .001
Latency Time (minute)	20.06 ± 7.33	21 ± 7.99	25 ± 7.85	31.44 ± 8.14*©¥	< .001
Sleep Freshness (VAS)	7.06 ± 1.48	6.88 ± 1.67	6.75 ± 1.95	6.31 ± 1.85	0.28
Spiegel Questionnaire (score)	25.94 ± 2.77	24.81 ± 2.71	22.88 ± 2.85*	22.06 ± 2.52*©¥	< .001

Min: minutes; VAS: visual analogue scale; COND 1: no blue light exposure; COND 2: blue light exposure from 7: 30 PM to 9: 00 PM; COND 3: blue light exposure from 9: 00 PM to 10: 30 PM; COND 4: blue light exposure from 10: 30 PM to 12: 00 AM.

* Significant difference compared to condition 1;

© Significant difference compared to condition 2;

¥ Significant difference compared to condition 3.

## RESULTS

### Subjective sleep measures

The results for sleep quality and quantity under the conditions No exposure, Exposure from 7: 30 PM to 9: 00 PM, Exposure from 9: 00 PM to 10: 30 PM, and Exposure from 10: 30 PM to 12: 00 AM are summarized in Table 1. The ANOVA revealed significant main effects of the timing of evening blue light exposure on the Spiegel Questionnaire score (F_3,45_ = 33.91, *p* < 0.001, ηp^2^ = 0.69 [*large])*, total sleep duration(F_3,45_ = 24.55, *p* < 0.001, *η*p^2^ = 0.62 [*large*]), and sleep onset latency (F_3,45_ = 14.29, *p* < 0.001, *η*p^2^ = 0.48 [*large*]). No significant effect of the timing of evening blue light exposure was observed on sleep freshness. The post-hoc test revealed a larger decrease in Spiegel Questionnaire scores for (i) the conditions Exposure from 9: 00 PM to 10: 30 PM and Exposure from 10: 30 PM to 12: 00 AM compared to the No exposure condition (*p* < 0.001), and (ii) the conditions Exposure from 9: 00 PM to 10: 30 PM and Exposure from 10: 30 PM to 12: 00 AM compared to the Exposure from 7: 30 PM to 9: 00 PM condition (*p* < 0.05 and *p* < 0.001, respectively) (Table 1).

Additionally, the post hoc test revealed a larger decrease in total sleep time for (i) the conditions Exposure from 9: 00 PM to 10: 30 PM and Exposure from 10: 30 PM to 12: 00 AM compared to the No exposure condition (*p* < 0.001), (ii) the conditions Exposure from 9: 00 PM to 10: 30 PM and Exposure from 10: 30 PM to 12: 00 AM compared to the Exposure from 7: 30 PM to 9: 00 PM condition (*p* < 0.05 and *p* < 0.001, respectively), and (iii) the condition Exposure from 10: 30 PM to 12: 00 AM compared to the condition Exposure from 9: 00 PM to 10: 30 PM (*p* < 0.05) (Table 1).

Regarding sleep onset latency, the post-hoc test showed significantly larger values recorded (i) in the condition Exposure from 10: 30 PM to 12: 00 AM compared to the No exposure condition, the condition Exposure from 7: 30 PM to 9: 00 PM, and the condition Exposure from 9: 00 PM to 10: 30 PM (*p* < 0.001, *p* < 0.001 and *p* < 0.05, respectively) (Table 1).

### Motor performance measures

The data related to motor performance are presented in Figure 1 for movement duration and in Figure 2 for dart-throwing accuracy.

The ANOVA showed significant main effects of the timing of evening blue light exposure on dart-throwing accuracy (F_3,45_ = 17.04, *p* < 0.001, *η*p^2^ = 0.51 [*large*]) and movement duration (F_3,45_ = 13.83, *p* < 0.001; *η*p^2^ = 0.47 [*large*]). These large effect sizes indicate substantial practical relevance for athletic performance, suggesting that evening blue light exposure timing could meaningfully impact athletes’ precision-based motor skills. The post hoc test revealed significantly longer movement duration in the Exposure from 9: 00 PM to 10: 30 PM and Exposure from 10: 30 PM to 12: 00 AM conditions compared to the No exposure condition (*p* < 0.001). Similarly, the post-hoc test showed a significantly longer time in the Exposure from 9: 00 PM to 10: 30 PM and Exposure from 10: 30 PM to 12: 00 AM conditions compared to the Exposure from 7: 30 PM to 9: 00 PM condition (*p* < 0.01 and *p* = 0.002, respectively) (Figure 1).

Regarding dart-throwing accuracy, the post-hoc test showed significantly lower accuracy scores (i) in the Exposure from 9: 00 PM to 10: 30 PM and Exposure from 10: 30 PM to 12: 00 AM conditions compared to the No exposure condition (p < 0.002 and *p* < 0.001, respectively), (ii) in the Exposure from 10: 30 PM to 12: 00 AM condition compared to the Exposure from 7: 30 PM to 9: 00 PM condition (*p* < 0.001), and (iii) in the Exposure from 10: 30 PM to 12: 00 AM condition compared to the Exposure from 9: 00 PM to 10: 30 PM condition (*p* < 0.05) (Figure 2).

### Cognitive performance measures

The summarized data on cognitive performance (selective attention) are presented in Figure 3. Statistical analyses (ANOVA) revealed a main effect of the timing of evening blue light exposure (F_2,26_ = 9.26,*p* < 0.001; *η*p^2^ = 0.38 [*large*]). The post-hoc analysis high-lighted significantly lower selective attention (i) in the Exposure from 9: 00 PM to 10: 30 PM and Exposure from 10: 30 PM to 12: 00 AM conditions compared to the No exposure condition (*p* = 0.003 and *p* < 0.001, respectively), and (ii) in the Exposure from 10: 30 PM to 12: 00 AM condition compared to the Exposure from 7: 30 PM to 9: 00 PM condition (*p* < 0.05).

## DISCUSSION

This study presents convincing evidence that the timing of exposure to blue light in the evening significantly impacts the quality of sleep, motor performance, and cognitive function in young athletes with intermediate chronotypes. This adds to the increasing amount of research on the disruption of circadian rhythms in athletic populations. The findings of our study suggest that exposure to blue light starting at 9: 00 PM has a negative impact on the structure of sleep, resulting in shorter sleep duration and longer time taken to fall asleep. The sleep disturbance has a detrimental effect on motor skills, as indicated by a decrease in accuracy when throwing darts and a longer movement time. It also impairs cognitive function, especially in tasks that need sustained attention. The results align with prior research, which has shown that exposure to blue light in the evening disrupts melatonin production, leading to a delay in the circadian rhythm and negatively affecting both physical and mental recovery processes [16, 19].

Our primary finding demonstrates that blue light exposure between 9: 00 PM and 10: 30 PM significantly reduced total sleep duration compared to earlier exposure and the control condition, aligning with the concept of the “critical window” for melatonin suppression proposed by Phillips et al. [39]. This result aligns with the research conducted by Burkhart and Phelps [40], which demonstrated that exposure to blue light within three hours before sleep can reduce both the quality and quantity of sleep by disrupting melatonin generation. The decrease in the duration during which our participants slept is most likely caused by the delayed release of melatonin, a critical component in regulating sleep [41]. These findings emphasize the susceptibility of the sleep-wake cycle to the timing of light exposure, especially in the hours before going to bed. Minimizing exposure to blue light at this time is necessary to preserve sufficient sleep length, which is vital for optimal physical recovery and sports performance.

Furthermore, our study found that exposure to blue light after 9: 00 p.m. affects sleep duration and significantly delays sleep onset. More precisely, participants exposed to blue light from 9: 00 PM to midnight experienced a delay in sleep onset compared to earlier exposure and the control condition. The findings support previous studies conducted by Chellappa et al. [42] and Silvani et al. [43], which showed that exposure to blue light in the evening can postpone sleep onset by increasing wakefulness and interfering with the body’s natural sleep-wake cycle. The delay in sleep onset seen in our study is likely caused by the increased alertness created by exposure to light in the late evening, which disrupts the body’s natural ability to fall asleep. The reported impacts on the time it takes to fall asleep and the length of sleep are especially significant for athletes, as achieving the best possible sleep is essential for physical recuperation, cognitive rejuvenation, and performance improvement [24]. The prolonged delay in falling asleep further reduces the total sleep time, thereby intensifying the adverse effects on recuperation and cognitive abilities. As a result, avoiding exposure to blue light during latenight hours is critical to promote prompt sleep initiation and improve the quality of restorative sleep.

Moreover, our findings suggest that the exposure to blue light during the time period from 9: 00 PM to midnight resulted in a notable decrease in cognitive performance, namely in the area of selective attention. This decline is particularly important for making decisions in sports and reacting quickly. This reduction was more pronounced compared to earlier exposure and the control condition. Hutton et al. [44] and Chang et al. [19] conducted prior research that found exposure to blue light in the evening negatively affects cognitive abilities, such as attention and alertness, on the following day. Our study attributed the decrease in selective attention to the disruptive impact of blue light on sleep quality, impacting cognitive performance. The decrease in selective attention that was seen is consistent with previous neuroimaging research that has demonstrated changes in activation patterns in the prefrontal cortex after exposure to blue light in the evening [45]. This may provide an explanation for the cognitive impairments observed in our investigation. While our findings align with research by Chang et al. [19] on blue light’s effects on cognitive function, they contrast with Hutton et al. [44], who observed less pronounced decrements in motor performance, potentially due to differences in age or activity levels in their population. Exposure to light during late-night hours likely disrupts sleep, diminishes cognitive acuity, and impairs concentration, both crucial for tasks requiring prolonged attention. These findings emphasize the importance of controlling evening light exposure for maintaining cognitive performance, especially in contexts where mental acuity is essential, such as in sports, academic work, or any high-performance environment.

To summarize, our research shows that exposure to blue light in the evening, especially after 9: 00 PM, has a significant negative impact on sleep patterns, motor skills, and cognitive performance in young athletes with intermediate chronotypes. This emphasizes the importance of maintaining good light hygiene to optimize athletic performance. Adverse effects are more noticeable when exposure occurs after 9: 00 PM, as this disturbs circadian rhythms and results in deficiencies the next day.

The findings of this study have significant (i) practical implications. Based on our findings showing 51% variance in dart-throwing accuracy and 47% variance in movement duration explained by blue light exposure timing, we strongly recommend implementing specific evening routines. The magnitude of these effects suggests that proper light management could provide a meaningful competitive advantage. Coaches, athletes, and sports health professionals should consider implementing strategies to limit screen time and blue light exposure before 9: 00 p.m. For instance, promoting the use of blue light filters or encouraging athletes to engage in alternative, low-stimulation activities in the hours leading up to sleep may help improve both physical and cognitive performance. Moreover, coaches and parents of adolescent athletes should consider establishing evening routines that limit screen time before bed, incorporating activities that promote relaxation and readiness for sleep. Educating athletes and their support networks about the benefits of minimizing blue light exposure in the hours before sleep could foster healthier habits that enhance both athletic and academic performance. Additionally, (ii) there are research implications; it is essential for researchers to consider the potential impact of evening blue light exposure, particularly in experiments conducted the following day that require optimal mobilization of cognitive functions, such as working memory, concentration capacity, and information processing speed, which are crucial in learning processes, such as solving complex problems and making strategic decisions. Reducing this exposure could not only enhance participants’ cognitive performance but also strengthen the validity of scientific results by minimizing biases caused by external disruptions to the circadian rhythm.

This study contributes to previous research suggesting that athletes and the general public should restrict their exposure to blue light in the evening to improve sleep quality and performance. Future research should focus on longitudinal studies to explore the long-term effects of consistent evening blue light exposure on sleep patterns and performance in adolescent athletes. Additionally, interventions such as the use of blue light-blocking glasses or digital device curfews should be investigated for their efficacy in improving sleep quality and athletic outcomes. Research should also examine varied sleep-wake rhythms, different chronotypes, and other exposure time frames to understand these interactions further.

## Limitations

This study elucidates the impact of evening blue light on young athletes; however, it has considerable limitations. The relatively small sample size of 16 male adolescent athletes with intermediate chronotypes restricts its generalizability to broader populations of young athletes. Future research should include a larger and more diverse sample to better understand the effects of blue light exposure across different populations. Additionally, our research focused on the immediate effects of blue light exposure over a brief duration. While this offers essential insights into immediate consequences, it does not account for long-term effects or adaptations resulting from prolonged exposure. Longitudinal studies could provide a deeper understanding of the lasting impacts of evening blue light exposure on athletes’ sleep and performance. In addition, potential confounders such as athletes’ training schedules and dietary habits were not controlled in this study and may have influenced the results. Future research should address these factors to better isolate the effects of blue light exposure.

## CONCLUSIONS

This study demonstrates that night-time exposure to blue light, particularly after 9: 00 PM, adversely affects sleep quality, motor performance, and cognitive function in young male athletes with intermediate chronotypes. The results correspond with prior studies on circadian rhythm disturbance in athletes and enhance our comprehension of the timing-dependent impacts of blue light exposure. The findings underscore the importance of regulating blue light exposure in the evening, especially after 9: 00 PM, to safeguard sleep quality and improve subsequent physical and cognitive function. These findings have implications not only for athletes but also for adolescents in general, particularly those balancing academic and extracurricular demands. Reducing evening blue light exposure may enhance cognitive performance and overall well-being, both in the sports context and beyond. Coaches, athletes, and sports health professionals should implement evidence-based strategies to minimize evening exposure to blue light. This can be accomplished by restricting screen time, employing blue light filters on electronic devices, and considering the utilization of blue light-blocking spectacles.

Furthermore, aligning daily activities with natural circadian rhythms may enhance recovery and optimize performance in competitive athletic settings. Additional research is required to assess the lasting effects of extended night-time blue light exposure on athletic performance and to explore potential interventions that may alleviate its negative impacts. Moreover, a study examining the relationship between chronotype, blue light exposure, and performance in other athletic disciplines could enhance the understanding of this phenomenon.
